# Prevention of amiodarone-induced cardiac toxicity in male BALB/c mice by a nutrient mixture

**DOI:** 10.3892/etm.2014.1518

**Published:** 2014-02-03

**Authors:** M. WAHEED ROOMI, NUSRATH WAHEED ROOMI, TATIANA KALINOVSKY, MATTHIAS RATH, ALEKSANDRA NIEDZWIECKI

**Affiliations:** Dr. Rath Research Institute, Santa Clara, CA 95050, USA

**Keywords:** amiodarone, nutrient mixture, creatine phosphokinase, aspartate aminotransferase

## Abstract

Amiodarone (Amio), a potent anti-arrhythmic drug, is associated with life-threatening pulmonary toxicity involving fibroses and inflammation. A unique nutrient mixture (NM) consisting of lysine, proline, ascorbic acid, *N*-acetyl cysteine and green tea extract has previously been shown to exhibit a broad spectrum of pharmacological, therapeutic, cardiovascular and chemopreventive properties. The present study was undertaken to determine whether the NM exhibits preventive effects on Amio-induced cardiac toxicity. Six-week-old male BALB/c mice were divided into four groups (A–D) of six animals per group. Mice in groups A and C were fed a regular diet for three weeks, while the diets of the mice in groups B and D were supplemented with 1% NM during that period. After three weeks, the mice in groups C and D received daily Amio injections of 50 mg/kg body weight intraperitoneally for 4 days, whilst those in groups A and B received saline alone. At 24 h after the final dose, mice were sacrificed, blood was withdrawn and serum was collected for clinical chemistry of the heart enzymes creatine phosphokinase (CPK) and aspartate aminotransferase (AST). In addition, livers, kidneys, hearts and lungs were excised and weighed. No significant differences in weight gain were identified among the groups and liver, kidney, heart and lung weights were comparable in all four groups. Administration of Amio to group C resulted in a significant increase in serum CPK levels, whereas in NM-fed group D, the CPK levels were comparable to those in the saline injection groups, A and B. Amio administration also resulted in a significant increase in serum AST levels in group C, but not in the group D animals which exhibited similar levels to those of groups A and B. Therefore, the results indicate that NM has the potential to protect against Amio-induced cardiac toxicity.

## Introduction

Amiodarone (Amio), an agent with vasodilatory and antiarrhythmic properties, has been used clinically in the treatment of ventricular arrhythmias, including recurrent ventricular tachycardia and fibrillation (1,2). Amio is typically administered intravenously or orally in daily high loading doses of 800–1,600 mg, until the arrhythmia is controlled or as daily maintenance oral doses of 200–600 mg for long term therapy. However, this iodine-containing compound tends to accumulate in several organs, including the lungs and has been associated with a variety of adverse events. Pulmonary toxicity is the most serious complication of Amio and usually manifests as acute or subacute pneumonitis (3,4). Liver toxicity appears to be more common with higher doses (5). Other adverse side-effects include fatigue, tremor, involuntary movements, poor coordination, peripheral neuropathy, nausea, vomiting, constipation, anorexia, visual disturbances, corneal deposits, skin discoloration and rash, photosensitivity, bradycardia and worsening of arrhythmias. Uncommon side-effects include pneumonitis, pulmonary fibrosis, optic neuropathy, blindness, thyroid dysfunction and liver injury (5).

Diverse antioxidants have been shown to prevent Amio-induced toxicity (6,7). A unique nutrient formulation (NM) containing primarily ascorbic acid, lysine, proline, *N*-acetyl cysteine and green tea extract has previously been shown to exhibit a broad spectrum of pharmacological, therapeutic, cardiovascular and chemoprotective properties (8). In previous studies, it was found that NM significantly inhibited acetaminophen-induced and carbon tetrachloride-induced hepatic and renal damage (9,10).

In the present study, the *in vivo* effects of the NM diet were examined in mice treated with Amio, focusing on cardiac enzyme levels.

## Materials and Methods

### Materials

Amio powder obtained from Sigma-Aldrich (St. Louis, MO, USA) was diluted in warm saline (pH 7.4) to 50 mg/ml. The stock solution of NM was composed of the following in the quantities indicated: 700 mg vitamin C (as ascorbic acid and as Mg, Ca and palmitate ascorbate); 1,000 mg L-lysine; 750 mg L-proline; 500 mg L-arginine; 200 mg *N*-acetyl cysteine; 1,000 mg standardized green tea extract (80% polyphenol); 30 μg selenium; 2 mg copper; 1 mg manganese; and 50 mg quercetin.

### Animals

Male BALB/c mice, free of murine viruses, bacteria and parasites, and ~6 weeks of age on arrival, were purchased from Simonsen Laboratories (Gilroy, CA, USA) and maintained in microisolator cages under pathogen-free conditions on a 12-h light/12-h dark schedule for 1 week. All animals were cared for in accordance with the institutional guidelines for the care and use of experimental animals.

### Experimental design

After one week of isolation, mice were divided into four groups (A–D) of six animals per group. Mice in groups A and C were fed a regular Purina mouse chow diet (Laboratory Rodent Diet 5001 from Purina Mills, LLC, purchased from Newco Distributing Inc., Rancho Cucamonga, CA, USA) for three weeks, while mice in groups B and D were fed the regular mouse chow diet supplemented with 1% (w/w) NM during that period. During the study, the mice consumed, on average, 4 g of their respective diets per day. Thus, the supplemented mice received ~20 mg NM per day. After three weeks, the mice in groups C and D received daily Amio injections of 50 mg/kg body weight intraperitoneally for 4 days and those in groups A and B received saline alone. The respective diets were continued for these 4 days. At 24 h after the final dose, mice were sacrificed, blood was withdrawn, serum was collected for clinical chemistry and livers, kidneys, hearts and lungs were excised and weighed.

### Serum analyses

Blood was collected and centrifuged at 13,000 × g for 5 min at 4°C. The samples were stored at −80°C until sent for analysis. Chemistry tests for serum creatine phosphokinase (CPK) and aspartate aminotransferase (AST) were run on a Hitachi 747 Chemistry Analyzer (Tokyo, Japan) with reagents from Boehringer Ingelheim (Ingelheim am Reine, Germany).

### Statistical analysis

Results are expressed as mean ± SD for each group. Data was analyzed by independent sample t-tests. MedCalc Software (Mariakerke, Belgium) was used. P<0.05 was considered to indicate a statistically significant result.

## Results

### Body weights and food consumed

No significant difference in weight gain was observed between the groups. The mean initial body weight of the mice was 24.1±1.4 g and the mean final weights were 26.2±0.7, 26.4±1.4, 27.6±2 and 26.7±1.9 g for groups A, B, C and D, respectively. The mean dietary intake for the supplemented groups was 3.5±0.5 g, 83% of the mean intake for the mice fed a control diet, which was 4.2±0.5 g (P=0.002).

### Vital organ weights

**Figure 1:**
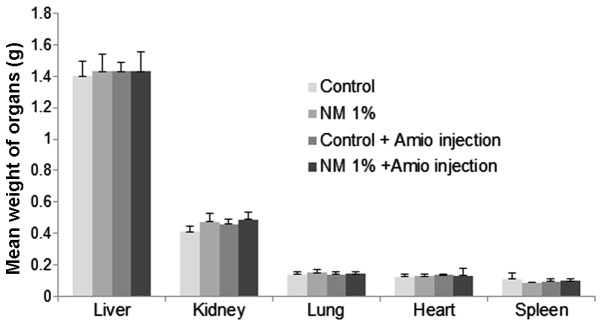
Effect of diet regimens and daily 50 mg/kg Amio treatment on the weight of vital organs in mice. Amio, amiodarone; NM, nutrient mixture.

Liver, kidney, heart and lung weights were comparable in all four groups, as shown in Fig. 1.

### Serum CPK

**Figure 2:**
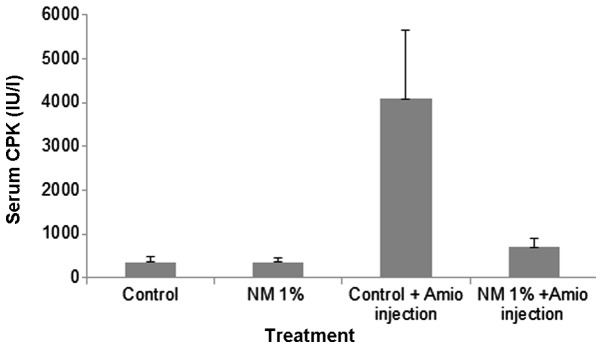
Effect of dietary regimens and daily 50 mg/kg Amio administration on serum CPK levels in mice. Amio, amiodarone; CPK, creatine phosphokinase; NM, nutrient mixture.

Administration of Amio to the control diet group resulted in a significant increase in the mean serum CPK level, whereas in the 1% NM-fed mice, the mean serum CPK following Amio administration was comparable to those in the saline injection groups. The serum CPK levels in the control and NM 1% groups (groups A and B) were 365±135 and 370±90 IU/l respectively. Treatment of the mice in group C with Amio resulted in a marked increase in the serum CPK level of 1,121% compared with that in group A and a mean CPK level of 4,090±1,560 IU/l. Supplementation with NM 1% prior to Amio administration, as performed with group D, resulted in a significantly reduced level of CPK. The mean CPK level of group D was 700±190 IU/l, a reduction of 83%, when compared with that in group C (P=0.001), as shown in Fig. 2.

### Serum AST

**Figure 3:**
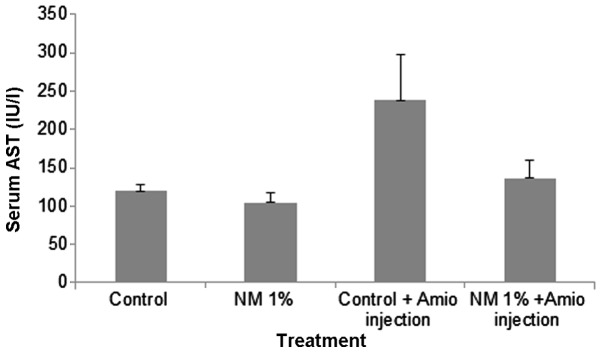
Effect of dietary regimens and daily 50 mg/kg Amio treatment on serum AST levels in mice. Amio, amiodarone; AST, aspartate aminotransferase; NM, nutrient mixture.

Amio administration also resulted in a significant increase in the serum marker for AST in the mice fed with the control diet, but not in mice fed with NM 1%, which exhibited similar levels to those of the saline injection groups, as shown in Fig. 3. Mean serum AST concentrations for groups A and B were 120±8 and 105±12 IU/l, respectively. Group C exhibited a marked increase with levels of 238±60 IU/l, which was a 198% increase compared with that of the control. Group D demonstrated a significantly reduced level of AST at 136±23 IU/l, which was a reduction of 43%, when compared with the level in group C (P=0.003).

## Discussion

The results of the present study demonstrate that pretreatment for three weeks with a diet supplemented with 1% NM reduced cardiac damage, as reflected in the enzyme levels of male BALB/c mice injected with daily toxic doses (50 mg/kg body weight) of Amio for four days. Amio treatment caused marked increases in cardiac serum CPK and AST levels in unsupplemented mice; however, supplementation with NM reversed the CPK and AST levels to near normal limits. The levels of CPK, an enzyme found in the heart, brain and skeletal muscles, increase with heart muscle damage; levels rise 4–8 h following an acute myocardial infarction, peaking at 16–30 h and returning to baseline within 4 days (11,12). AST, an enzyme that is normally present in heart and liver cells, is released into the blood when the liver or heart is damaged. The amount of AST in the blood is directly associated with the extent of tissue damage (11).

A study of the role of free radicals in the toxicity of Amio by Verikei *et al* revealed that Amio generates free radicals, under *in vitro* and *in vivo* conditions, that may play a role in the pathogenesis of Amio toxicity, as well as in other well-established mechanisms. The study also revealed that antioxidants may have a partial protective effect against Amio toxicity (13). Vitamin C has been shown to decrease Amio-induced toxicity in rat thymocytes by restoring cellular glutathione content (7). The antioxidants vitamin C and *N*-acetyl cysteine were shown to protect mouse fibroblasts from Amio-induced cytotoxicity (6). In a literature review, Harling *et al* found that vitamin C and E significantly decreased postoperative atrial fibrillation (14).

The NM tested in the present study was formulated based on the targeting of various physiological processes involved in a wide spectrum of pathological conditions at the cellular level. Based on our own studies and published data, it was hypothesized that metabolic effects are likely to result from the synergy of ascorbic acid, lysine, proline, green tea extract, arginine, *N*-acetyl cysteine, quercetin, selenium, copper and manganese. Combining these micronutrients expands metabolic targets, maximizing biological impact with lower doses of components. A previous study of the comparative effects of NM, green tea extract and epigallocatechin gallate (EGCG) on the inhibition of MMP-2 and MMP-9 secretion in various cancer cell lines with varying MMP secretion patterns, revealed the superior potency of NM over green tea extract and EGCG at equivalent doses (15).

In conclusion, the present study demonstrated that pretreatment for three weeks with a diet supplemented with 1% NM reduced the cardiac damage in BALB/c mice caused by the administration of multiple toxic doses of Amio. Supplementation with dietary NM reduced the Amio-induced elevated cardiac enzymes in the mice. Although clinical studies are required, the results indicate the therapeutic potential of using NM adjunctively with Amio to protect against Amio-induced heart damage.
